# What makes primary care effective for people in poverty living with multiple chronic conditions?: study protocol

**DOI:** 10.1186/1472-6963-10-320

**Published:** 2010-11-30

**Authors:** Christine Loignon, Jeannie L Haggerty, Martin Fortin, Christophe P Bedos, David Barbeau, Dawn Allen

**Affiliations:** 1 Centre de recherche de l'Hôpital Charles LeMoyne, Université de Sherbrooke (150 Place Charles Lemoyne), Longueuil (J4K 0A8), Canada; 2 Department of Family Medicine, St-Mary's Hospital, McGill University (3777 Jean Brillant), Montréal, (H3T 1M5), Canada; 3 Faculty of Dentistry, McGill University (3550 University), Montréal (H3A 2A7) Canada; 4 Department of Family Medicine, Université de Montréal (2900 Édouard Montpetit), Montréal (H3T 1J4), Canada; 5 Programs in Whole Person Care, McGill University (1650 Cedar Avenue), Montréal (H3G 1A4), Canada

## Abstract

**Background:**

The inverse care law persists: people living in poverty have the greatest needs and face considerable challenges in getting the care they need. Evidence reveals that GPs encounter difficulties in delivering care to poor patients, while many of those patients feel stigmatized by healthcare professionals. Patients living in poverty report negative healthcare experiences and unmet healthcare needs. Indeed, there is a growing recognition in primary care research of the importance of addressing the capabilities and social conditions of the poor when delivering care. Few studies have looked at the factors contributing to effective and "socially responsive" care for people living in poverty.

**Methods/Design:**

Our study adopts a qualitative ethnographic approach in four healthcare organizations in deprived areas of metropolitan Montreal (Québec, Canada), using patient shadowing techniques and interviews. Data will be collected through fieldwork observations and informal interviews with patients before and after consultations. We will observe medical consultations, care organization activities, and waiting areas and reception of patients. We will conduct a total of 36 individual interviews with 12 GPs and 24 patients. The interviews will be audio-recorded and transcribed for purposes of analysis. The analysis consists of debriefing sessions, coding and interpretive analysis.

**Discussion:**

This study aims to investigate how positive healthcare interactions between physicians and patients can improve the management of chronic conditions. We hypothesize that factors related to care organization, to healthcare professionals' experience and to patients may enhance the quality of healthcare interactions, which may have positive impacts for preventing and managing chronic conditions. Our study will provide a unique set of data grounded in the perspectives of healthcare professionals and of patients living in poverty.

## Background

Most studies on care interactions between physicians and people living in poverty have provided descriptive data mainly on problems and challenges. Health professionals, physicians or dentists, spend less time with low-income patients and find it difficult to deal with their non-compliance with treatments [1-4]. Likewise, people living in poverty are more likely to postpone medical visits and to manage their health as a series of crises [5]. Some of these problems are related to the social distance between health professionals and people living in poverty,[6,7] but few studies have explored this avenue of research.

Consequently, studies on care interactions and interpersonal relationships in healthcare do not offer any in-depth understanding of how these interactions unfold. Taking into account the capabilities and resources of people living in poverty contributes to positive interactions between dentists and patients and may help healthcare professionals provide effective care [8]. Unfortunately, studies in primary care to date provide no data on what helps make these interactions positive and how some physicians develop best practices in this regard. It is therefore essential, given the vulnerability, both social and health-related, of people living in poverty, to obtain contextualized data on positive healthcare interactions that support the management of chronic conditions. Thus, our study's objectives are to investigate: 1) what factors contribute to positive care interactions between physicians and patients, and 2) how these factors can help improve the management of chronic conditions. Achieving these objectives can contribute to the development of professional training and innovative medical practices that take into account peoples' conditions of poverty [9].

### Poverty and chronic illness

It is well-known that living in poverty increases the risk of developing a chronic illness [10,11]. According to the Canadian Community Health Survey, among Canadians with the lowest incomes, 40% suffer from chronic illnesses [12]. People living in households with incomes under $20,000 are three times more likely to experience a decline in health status than those at higher income levels [13]. Compared to those who are more well-off, poor people have more health problems associated with chronic illnesses and suffer more psychological distress, all the while experiencing difficulty in obtaining care that is responsive to their needs [14]. According to studies by Fortin and colleagues, multimorbidity is associated with psychological distress and the perception of socio-economic status [15]. Moreover, these studies have also revealed relationships between multimorbidity and quality of life, family income and the perception of socio-economic status [16].

The harmful health-related behaviours of the poor (smoking, poor nutrition, etc.) are well-known. Nevertheless, some researchers suggest studying the effects of social processes and structural constraints such as exclusion, discrimination and the sense of powerlessness on health behaviours and the use of healthcare [17,18]. It may be that such structural conditions and forces have a greater potential impact than health behaviours when it comes to explaining the poor health status of persons living in poverty. Negative healthcare interactions in which people feel stigmatized may impede prevention and management of chronic conditions in these populations. Recognizing and addressing the influences of social constraints on health behaviour change and the adoption of optimal chronic illness self-care strategies among patients is an important dimension of effective patient-centered chronic care.

### Chronic illnesses and primary care

Chronic illnesses are the primary cause of death in industrialized countries [19]. According to the WHO report, *Innovative Care for Chronic Conditions: Building Blocks for Action*, 46% of deaths worldwide are due to chronic illnesses [20]. This is true, as well, for the province of Quebec (Canada), where chronic illnesses are the principal cause of death, according to the *Institut national de santé publique du Québec *[Quebec Public Health Institute] [21]. As well, there is increasing recognition of the phenomenon of multimorbidity, which complicates the management of illness. In a recent study conducted in primary care practices, the average number of health problems was 2.8 among those aged 18-44, 4.6 among those aged 45-64, and 6.4 for those aged 65 and older. Nine out of 10 patients had more than one chronic health problem, and half had more than five [22].

Chronic illnesses present major challenges for both care providers and healthcare systems. In the United States, chronic illnesses are the primary reason for using healthcare services and account for 70% of healthcare spending [23]. According to Holman and Lorig, healthcare systems should support and strengthen the relationship between physician and patient in the treatment and follow-up of chronic illnesses. The effectiveness of healthcare depends on a high level of quality of care on the part of professionals, but also on patients' involvement in managing their chronic illness. Patients' self-management requires that they be able to obtain services based on a solid therapeutic alliance with their physician. Yet people living in poverty experience difficulties in their interactions with physicians that hinder the establishment of a therapeutic alliance. These people are most often dissatisfied with their medical visits [24,25]. As for physicians, they also find it difficult to establish a therapeutic relationship with patients who are living in poverty.

### Studies' limitations and avenues for research

While the social sciences have made important contributions to the study of healthcare interactions, there has been little progress in recent years, and the theoretical approaches are outdated, that is, they do not correspond to the new realities in which relationships between patients and healthcare professionals are formed today (collaborative care, shared decision-making, etc.)[26] More specifically, with respect to interactions between physicians and their patients, most studies have focused on relational problems and on deficiencies in healthcare effectiveness. A few studies, however, have looked at some promising approaches for improving interactions between physicians and patients. Thus, there are studies that stress the importance of a "patient-centred" model of care that would reduce patients' dissatisfaction, improve their sense of control, reinforce their compliance with treatment and improve their health status [27]. Nevertheless, these studies have certain limitations. In terms of methodology, they are most often based on quantitative approaches that do not allow for in-depth investigation of patients' and physicians' lived experience and perspectives on factors that support therapeutic interactions. In terms of populations studied, the research does not look specifically at people living in poverty, such that we do not know what these people consider to be positive in therapeutic interactions.

### Links with our previous and current research

We recently undertook a study of dentists which showed that some dentists had developed knowledge about the impacts of their patients' living conditions on their health and use of dental services [28]. These dentists practise in underprivileged areas and have developed an approach we call "humanistic" [8]. The positive impacts of this humanistic approach, on both the clinical and human levels, were reported to us by dentists who had participated in one of our studies. One of the inherent limitations of that study was that we were not able to observe this approach and the context in which it takes shape. To generate knowledge about the context in which these interactions occur, we conducted an ethnographic project in a dental practice located in one of the poorest neighbourhoods of Montreal. (Loignon et al., 2010) The data from the investigator's observations in the clinic enriched the data obtained from the interviews. In fact, being on-site at the clinic allowed us to observe the clinical environment in which this humanistic approach is formed and to validate some of our hypotheses. We observed how the professionals (dentist and assistant) accommodated the proposed treatments to the needs and expectations of low-income patients. We noted that this helped put patients at ease and gave them a positive sense of control. The professionals considered that this way of interacting with patients strengthened the therapeutic alliance.

We feel that these results are of great relevance for primary care interventions among people experiencing poverty and living with chronic illness. In fact, the patient's partnership with the physician in the self-management of chronic illnesses is key to a successfully managed treatment plan, and this is not possible without a therapeutic physician-patient alliance. A solid therapeutic alliance is fundamental to continuity of care [29]. A synthetic review of studies carried out in Canada and the United Kingdom, done within the scope of priority funding for continuity of care, showed that patients' involvement in their own care plays a major role in maintaining a therapeutic alliance and promoting continuity of care, but that a certain proportion of patients-those who are poor-lack adequate resources or capacities for effective self-management [30]. These patients would therefore be more vulnerable to interruptions in service and more dependent on care providers.

### Research objectives

The aim of this research is to identify and understand the factors that contribute to effective delivery of care for people living in poverty with multiple chronic conditions. More specifically, we propose to identify the factors that contribute to positive interactions between primary care doctors and patients living in poverty, and to understand the impacts these factors have on the management of chronic conditions.

## Methods/Design

This qualitative study uses ethnographic methods to examine doctor-patient interaction at primary care clinics in deprived areas of metropolitan Montreal, in Canada. Ethnography, which is little used in primary healthcare research, is a research methodology that is useful when a subject calls for in-depth investigation from different perspectives. We know very little about the factors that promote positive interactions between primary care physicians and people living in poverty, especially in relation to the management of chronic conditions. Ethnographic methods will allow us to immerse ourselves in the environments, observe real-time interactions and examine the different emergent contextual dynamics. Having a member of the research team on-site will make it easier to recruit physician-patient dyads and to observe the clinical environment and how patients are received. These observational data will enrich and validate the content of the interviews.

### Study population and sampling

The target population consists of family physicians whose clientele includes a significant proportion of people living in poverty and adults living in poverty who have chronic illnesses and consult primary care physicians.

In this study, we will work in four medical clinics. These sites have been selected because they represent different structures providing primary care services. As well, because all four are located in the most disadvantaged neighbourhoods of metropolitan Montreal, these organizations provide care to vulnerable populations (poor and with multiple co-morbidities). These sites have also developed an expertise in providing care to vulnerable populations. Because we are interested in organizational and relational factors that promote positive care processes, this expertise adds to these sites' relevance for our research. From these four sites, we will select our sample of patients and physicians using a "purposive" sampling approach [31].

In selecting family physicians at these four clinics, we will apply the following criteria: they regularly provide care to low-income patients who have at least one chronic illness; they work at least one day a week at the clinic, and they have a significant proportion of low-income patients (10% to 20%). We will exclude physicians who mostly treat acute conditions or look after "walk-in" patients.

Likewise, in selecting patients who have visited a physician at one of these four clinics, we will apply several criteria: they are between 25 and 65 years old; they receive employment insurance benefits or have a family income below the low-income threshold (Statistics Canada's below-tax low-income threshold); they have had two or more chronic illnesses for more than a year; they can express themselves easily in French; and they are followed by a physician participating in the study. The age criterion was set thus because we want to target people who are potentially active and have already been treated for chronic illnesses. We also wish to avoid people in an older age bracket, who constitute a very different clientele in terms of healthcare services.

We plan to recruit 36 people: 12 physicians and two patients for each. We will thus obtain 24 physician-patient dyads. This dyad approach will allow us to validate information from different sources and also to identify divergences and convergences among viewpoints on therapeutic alliance. Based on our previous studies, we believe that 36 interviews will be enough to obtain the information required and to achieve theoretical generalization. We should, however, add that sample size will be determined by the principle of saturation, so there could be some slight variation from the target of 36 participants.

### Data collection

#### Phase 1: Observation process

Phase 1 will consist of making contact with each of the clinics and doing the observations. This phase will be carried out successively, one clinic at a time, according to the convenience of the clinic's professionals. At each of the four clinics, the investigator (Loignon) and the research assistant will first attend a team meeting to meet the professionals and present the research project. Attending this meeting will be part of the ethnographic process, as we will take observation notes on what happens during this activity. As well, we will conduct observations of the clinic, especially the reception and waiting areas, with the aim of immersing ourselves in the environment and observing patients' reception and the interactions between professionals and patients. The research assistant will conduct observation days at each of the clinics. They will be planned in accordance with the physician-patient dyad recruitment procedure. We plan to allocate a maximum of four weeks per clinic, at three to seven hours per day, depending on the clinic's activities and physicians' schedules. This observation period will be conducted in parallel with the recruitment of our physician-patient dyads.

We will keep a log of this process. Keeping a log throughout the whole period of the ethnographic process is recommended in order to prepare syntheses to be used in the analyses of observations or discussions in the field and of the interviews [32]. Entries will be made in this log on-site, and it will remain with the research assistant at all times. In addition, for each clinic, summary sheets will be prepared with data from the research assistant's notes in this log. These summaries will be integrated into the data analysis procedure.

#### Phase 2: Interview process

Phase 2 will consist of semi-structured individual interviews with the physicians and patients. Phase 2 will closely follow phase 1, but we expect to conduct the interviews within the first 22 months. These face-to-face interviews with every participant-both physicians and patients-will be carried out by the research assistant, who will use interview guides prepared for each type of participant. Interviews will last approximately one to two hours and will be conducted in a quiet space suitable for confidential conversation. They may take place in the clinic or at home, according to each participant's preference. The research assistant will present the study and will have the participant read and sign the consent form. These interviews will be audio-recorded and transcribed. After each interview, the research assistant will complete a summary interview report in which he will note any recommendations for future interviews (questions to be refined, topics to be added, etc.). Also, after each interview, the research assistant and one of the investigators (Loignon) will review and discuss the interview.

#### Phase 3: Data analysis and interpretation

The data will be organized[33] using coding that will consist of labelling, from each transcription and each summary sheet, the different elements touched upon in the interviews or emerging from the observations. We will create a summary list of codes for the different variables under study. This list will be developed during the course of the analysis as new codes are inferred and added and pre-existing codes refined. The results will then be presented in tables that will summarize the data gathered for each of the 24 dyads and for each series of six dyads per clinic.

Also, working together from these tables, the research team will develop and verify conclusions. Our methodological approach will be characterized primarily by near-simultaneous data collection and analysis and by a recursive analytical process. Thus, each interview will be coded as soon as it has been transcribed, and the data will be immediately entered into the tables. Interpretations drawn from the tables may provide feedback into other phases of the analysis. Data collection and analysis will continue in this way until data saturation is achieved and the conclusions are stable. We will used NVivo (QSR) software to support the analyses. Triangulation procedures will be employed at all stages of the study to ensure the validity of the analyses and interpretations. We will also use triangulation in formulating hypotheses and developing conclusions. To this end, the team members will meet regularly to interpret the findings.

Finally, our study meets the criteria for results transferability, which is a hallmark of the qualitative research approach [34]. The detailed description of each stage of our multiple-case study will make it possible to transfer the results obtained and apply them to other similar contexts [35]. Moreover, because the main advantage of the ethnographic approach is that it provides a deeper understanding of one or more cases, it is useful for generating questions and hypotheses that can then be explored using other methodologies [36].

### Ethical considerations

This study is based on the usual ethical principles, such as every person's right to refuse to participate in the study and to withdraw at any time, as well as respect for all participants and protection of their privacy. Each person recruited will receive all the information necessary to provide free and informed consent. We will guarantee to the participants that our records will be kept in the strictest confidence, and we will ensure the confidentiality of all statements by assigning identification numbers to all participants to protect their identities. The records and audio recordings will be kept, sealed, at the *Hôpital Charles LeMoyne *(HCLM) research centre and will be destroyed at the end of five years. No names will appear on any public documents and we will take every precaution to divulge no information that would allow a third party to identify a participant. These ethical principles are clearly stated on the consent form approved by the Committee for Ethics and Research of the *Hôpital Charles LeMoyne *(HCLM).

## Discussion

This study is one project (Project 2) in a research program aimed at improving the adequacy of healthcare for people living in poverty. This program includes two other research projects, one of which is funded by the Canadian Institutes of Health Research (CIHR 200361); the three projects constitute a comprehensive whole. Through a series of in-depth interviews with general practitioners, the first project (Project 1) proposes to identify the dimensions of the social competence process that supports the creation of a therapeutic relationship between physicians and patients living in poverty with at least one chronic illness[37].

Identifying the dimensions of the social competence process will be very useful to frame our direct observation of interactions between physicians and their low-income patients, which is the first phase of the project developed in this research protocol. As explained, the second project (Project 2) involves in-clinic observations of the interactions between physicians (and other health professionals with whom they work) and low-income patients, as well as interviews. Such ethnographic fieldwork methods will allow us to observe from a holistic perspective the subtleties of the doctor-patient relationship, allowing us to deepen considerably our understanding of such a complex relationship. Finally, a third project (Project 3) will consider the perspective of people living in poverty. This community-based participatory action research project will identify, from their perspective, what solutions people living in poverty might envision to help them better navigate the healthcare system.

Residents and professors in family medicine will be informed of the results of our study. We will work in collaboration with our colleagues in the Department of Family Medicine at the *Université de Sherbrooke *(Québec, Canada) to enrich the teaching module on communication with patients by providing data on techniques or approaches that can improve the therapeutic alliance with patients living in poverty. This project is conducted in partnership with the Montérégie Regional Health Authority. Our results will be directly transferred to decision-makers involved in the regional chronic disease monitoring program called COMPASS. Finally, we will contact low-income populations and invite them to participate in the research process. We will invite representatives of these groups to sit on an advisory committee that will meet quarterly. At these meetings, we will discuss the research themes that have been developed and future orientations proposed by the committee members. The purpose of these meetings will be to encourage input from different populations to broaden our research agenda and increase its societal influence.

This study will produce concrete recommendations for developing health interventions that address the needs and expectations of people living in poverty. Health inequalities and disparities in healthcare services are two major issues at the heart of health policy development, at both the national and international levels. In fact, one of the major issues that emerged from recent consultations carried out in Canada on healthcare services was that of ensuring access for everyone to services focused on patients' needs [38]. In addition, our study responds to the recommendations of a working group sponsored by the Canadian Institute for Health Information and the Canadian Population Health Initiative, calling for closer investigation of the lived experience of poor people and better understanding of what can be done to lessen the impacts of poverty, and also pointing out that there are major gaps in research on the relationship between poverty and health [39]. Ultimately, our aim is to strengthen the capacity of the healthcare system and of professionals to provide care that is adapted to the social conditions of people living in poverty.

## Competing interests

The authors declare that they have no competing interests.

## Authors' contributions

CL conceived the study, drafted the manuscript and coordinates the data collection and analysis. JL, MF and CB participated in the design of the study and participate in the qualitative analysis. DB participated in the design of the study and is involved in the collection of data. DA participates in the analysis of the data. All authors read and approved the final manuscript.

## Pre-publication history

The pre-publication history for this paper can be accessed here:

http://www.biomedcentral.com/1472-6963/10/320/prepub
